# Preoperative magnetic resonance imaging-radiomics in cervical cancer: a systematic review and meta-analysis

**DOI:** 10.3389/fonc.2024.1416378

**Published:** 2024-07-04

**Authors:** Linyong Wu, Songhua Li, Shaofeng Li, Yan Lin, Dayou Wei

**Affiliations:** Department of Medical Ultrasound, Maoming People’s Hospital, Maoming, Guangdong, China

**Keywords:** cervical cancer, radiomics, deep stromal invasion, lymphatic vascular invasion, lymph node metastasis

## Abstract

**Background:**

The purpose of this systematic review and meta-analysis is to evaluate the potential significance of radiomics, derived from preoperative magnetic resonance imaging (MRI), in detecting deep stromal invasion (DOI), lymphatic vascular space invasion (LVSI) and lymph node metastasis (LNM) in cervical cancer (CC).

**Methods:**

A rigorous and systematic evaluation was conducted on radiomics studies pertaining to CC, published in the PubMed database prior to March 2024. The area under the curve (AUC), sensitivity, and specificity of each study were separately extracted to evaluate the performance of preoperative MRI radiomics in predicting DOI, LVSI, and LNM of CC.

**Results:**

A total of 4, 7, and 12 studies were included in the meta-analysis of DOI, LVSI, and LNM, respectively. The overall AUC, sensitivity, and specificity of preoperative MRI models in predicting DOI, LVSI, and LNM were 0.90, 0.83 (95% confidence interval [CI], 0.75-0.89) and 0.83 (95% CI, 0.74-0.90); 0.85, 0.80 (95% CI, 0.73-0.86) and 0.75 (95% CI, 0.66-0.82); 0.86, 0.79 (95% CI, 0.74-0.83) and 0.80 (95% CI, 0.77-0.83), respectively.

**Conclusion:**

MRI radiomics has demonstrated considerable potential in predicting DOI, LVSI, and LNM in CC, positioning it as a valuable tool for preoperative precision evaluation in CC patients.

## 1 Introduction

Uterine cancer remains a prevalent public health challenge that poses a significant threat to women’s well-being globally. The latest cancer statistics in the United States anticipate 66,200 new cases in 2023, ranking third among gynecological malignancies, with an expected 13,030 fatalities, ranking sixth (1). The most common types of uterine cancer are endometrial cancer (EC) and cervical cancer (CC). Deep stromal invasion (DOI), lymphatic vascular space invasion (LVSI) and lymph node metastasis (LNM) are pivotal factors that influence preoperative therapeutic planning and the determination of postoperative adjuvant treatment strategies for uterine cancer. Specifically, DOI is defined as stromal invasion depth exceeding one-third of the myometrial thickness, including parametrial invasion. For example, the expert consensus meetings divided uterine cancer into four risk levels based on histology, grade, stage, and the presence of LVSI, aiming to reflect the likelihood of tumor invasion and recurrence and, thereby guiding potential adjuvant therapy (2, 3). Notably, patients without DOI could choose less aggressive surgery to mitigate perioperative and postoperative complications. Conversely, those suspected of having DOI often require combined chemoradiotherapy following surgery (4). Patients with LVSI positive uterine cancer may still have distant metastasis despite receiving adjuvant treatment (5). A study involving 368 uterine cancer patients revealed that 70% of them underwent unnecessary lymphadenectomy, even among high-risk populations, such as those with tumors larger than 2 cm and evidence of DOI (6). In this context, DOI, LVSI, and LNM determined the patient’s surgical plan and choice of adjuvant therapy through precise preoperative evaluation of the uterine cancer.

Magnetic resonance imaging (MRI) serves as a pivotal tool for preoperative evaluation of uterine cancer. Preoperatively, MRI can facilitate the identification of DOI. A study revealed that a radiologist with 7 years of experience in gynecological cancer imaging achieved a diagnostic sensitivity (SENC) of 70%, while a radiologist with 4 years of experience only attained a SENC of 50% (7). However, microscopic parametric invasion could be detected in approximately 32–36% of IB2/IIA patients, which often fell within a microscopic field that may be undetectable by radiologists (8). LVSI primarily involves microscopic-level analysis, which poses challenges for visual evaluation. Although MRI utilizing quantitative apparent diffusion coefficient has been explored for LVSI assessment, the results have been unsatisfactory (9). In assessing LNM, radiologists primarily rely on visual imaging to analyze the size, morphology, internal structure, and enhancement pattern of lymph nodes (10). For example, swollen lymph nodes, with a nearly circular shape, and irregular edges were common visual signs that were suspected of metastasis. However, similar visual characteristics can also be attributed to inflammatory changes, and postoperative pathological analysis reveals that only 50% of such lymph nodes are truly metastatic (11). Furthermore, some scholars had calculated that the SENC of preoperative MRI for normal sized LNM was only 0.59, with the area under the curve (AUC) of 0.70. Puncture pathology is an important tool of preoperative acquisition for DOI, LVSI, and LNM. However, since puncture is an invasive method, it may not only lead to complications such as tumor dissemination, but also increase the fear and resistance of patients. In addition, due to tumor heterogeneity, biopsy results may yield false-negative outcomes (12, 13). This underscores the urgent need to transcend visual limitations and harness novel technologies that capture more comprehensive tumor image information to achieve precise preoperative evaluation of uterine cancer.

Accurate preoperative identification of DOI or LVSI or LNM in patients with uterine cancer holds significant implications for treatment management, effectively averting the pitfalls of over- and under-treatment. Radiomics (R), an artificial intelligence (AI) technology, transcends the limitations of visual inspection by leveraging computers to quantify microscopic image features, thereby enhancing the utilization of image information and advancing towards precision medicine. Compared to traditional imaging diagnosis, R is capable of reflecting tumor biological characteristics in a more comprehensive and detailed manner, while being unaffected by specimen size, location, or quality, thus better capturing tumor heterogeneitym, including molecular genetic alterations. R has garnered widespread application in preoperative risk assessment of uterine cancer (EC and CC). For example, the R model developed based on T2WI and DWI images had been validated to have SENC and specificity (SPEC) of 0.60 and 0.96 for predicting the LVSI state of early CC (14); the R model based on MRI images of 339 EC patients from 5 centers was developed to predict LVSI with SENC and SPEC of 0.92 and 0.74, respectively (15); the SENC and SPEC of the R model based on enhanced T1WI and T2WI images in predicting the LNM state of early CC were validated to be 0.71 and 0.72, respectively (16). While R has exhibited superior detection performance compared to clinical models (C), the integration of radiomics-clinical models (R-C) offers even greater clinical benefits. For example, the nomogram jointly developed based on R features, MR reported LN status, and the International Federation of Gynecology and Obstetrics (FIGO) stage was superior to both single R model and C model (17). Based on these findings, the predictive potential of R in forecasting DOI, LVSI, and LNM of EC was further validated through meta-analysis. For example, a meta-analysis encompassing 15 studies revealed that the overall SENC and SPEC of R for predicting DOI, LVSI, and LNM were 0.74 and 0.82; 0.66 and 0.75; 0.78 and 0.81, respectively (18). However, only a meta-analysis for CC in terms of LNM has been reported, necessitating further analysis for CC in DOI and LVSI. Additionally, the potential value of R-C models remains unexplored, requiring further meta-analysis to uncover its promise.

The purpose of this systematic review and meta-analysis was to evaluate the considerable potential of R developed from preoperative MRI, in detecting DOI or LVSI or LNM in CC. In addition, the potential based on C, and R-C models in CC was also analyzed, thereby providing insights into the efficacy of these approaches in improving diagnostic accuracy and clinical decision-making.

## 2 Methods

### 2.1 Search scheme

A systematic search was undertaken in the PubMed database, encompassing original studies published up to March 31, 2024. The search was guided by a set of keywords including “Radiomics”, “Texture”, “Cervical cancer”, “Parametrial invasion”, “Stromal invasion”, “Lymphovascular space invasion”, “Lymphatic vascular space invasion”, “LVSI”, “Lymph node metastasis”, and “LNM”. Two reviewers with more than 3 years of experience in abdominal imaging diagnosis independently reviewed the original study of preoperative R, including the study abstract and full text. Discrepancies between reviewers were resolved by consensus or, in cases of persistence, by a third reviewer with over five years of abdominal imaging diagnosis expertise. In order to compare the performance of different models, R, C, and R-C models were included separately. It is noteworthy that the C model was exclusively incorporated in studies that had an underlying R cohort.

### 2.2 Study selection

Inclusion criteria for literature selection: (1) original MRI-based R/texture feature analysis studies. (2) patients with CC confirmed by histopathological examination. (3) preoperative prediction for DOI or LVSI or LNM. (4) availability of data, including true positive (TP), false positive (FP), true negative (TN) and false negative (FN), for calculation purposes.

The exclusion criteria were as follows: (1) R studies based on non CC lesion images. (2) R studies using CT, PET/CT, and ultrasound (US). (3) comments, meta-analyses, case reports, guidelines or errata, repeated studies. (4) postoperative R studies. (5) studies involving preoperative anti-tumor treatment; (6) deep learning studies; (7) Radiomics Quality Score (RQS) of 10 or below.

### 2.3 Data extraction

The literature data were extracted from the original studies: (1) basic characteristics, such as the author, publication year, country, and study design. (2) cohort characteristics, including the cohort type, sample size, and the population specific to DOI or LVSI or LNM. (3) image characteristics, comprising the image protocol, image segmentation, R extraction software, feature selection strategy, and model algorithms. (4) evaluation indicators, namely the AUC, SENC, SPEC, and the Delong test. The numbers of TP, TN, FP, and FN were calculated according to the SENC and SPEC in each study report, referred to the formula: SENC=TP/(TP+FN), SPEC=TN/(FP+TN). In cases where multiple models were based on the same cohort, the model with superior performance was included.

### 2.4 Study quality assessment

The RQS was used to evaluate R quality, which was an important tool to measure the rigor of artificial intelligence (AI) study. RQS included 16 evaluation indexes, covering aspects such as image acquisition, image preprocessing, validation, performance evaluation, practicality, open science (19). Additionally, the Quality Assessment of Diagnostic Accuracy Studies (QUADAS-2) also was utilized to evaluate the methodological quality, including: (1) patient selection, (2) index test, (3) reference standard, and (4) flow and timing. The risk of bias in each category was classified as low, high, or unclear. A modified version of QUADAS-2 proposed by Sollini et al. and validated by Bedrikovetski S et al. was utilized (20, 21).

### 2.5 Statistical analysis

Using the Stata software (version 12.0), a comprehensive analysis was conducted to summarize and calculate the TP, TN, FP, and FN. Forest plots were generated to visually represent the overall SENC and SPEC across studies. The Cochrane diagnostic test and I^2^ statistic were employed to evaluate heterogeneity among the studies, with I^2^ values exceeding 50% indicating high heterogeneity (22). Deek’s funnel plots were utilized to evaluate whether the analysis was subject to publication bias. The summary receiver operating characteristic (sROC) curve demonstrated the predictive potential of R studies. MetaDiSc software was utilized for subgroup analysis (23). A statistical significance level of P < 0.05 was set for all analyses.

## 3 Results

### 3.1 Results of search scheme

**Figure 1:**
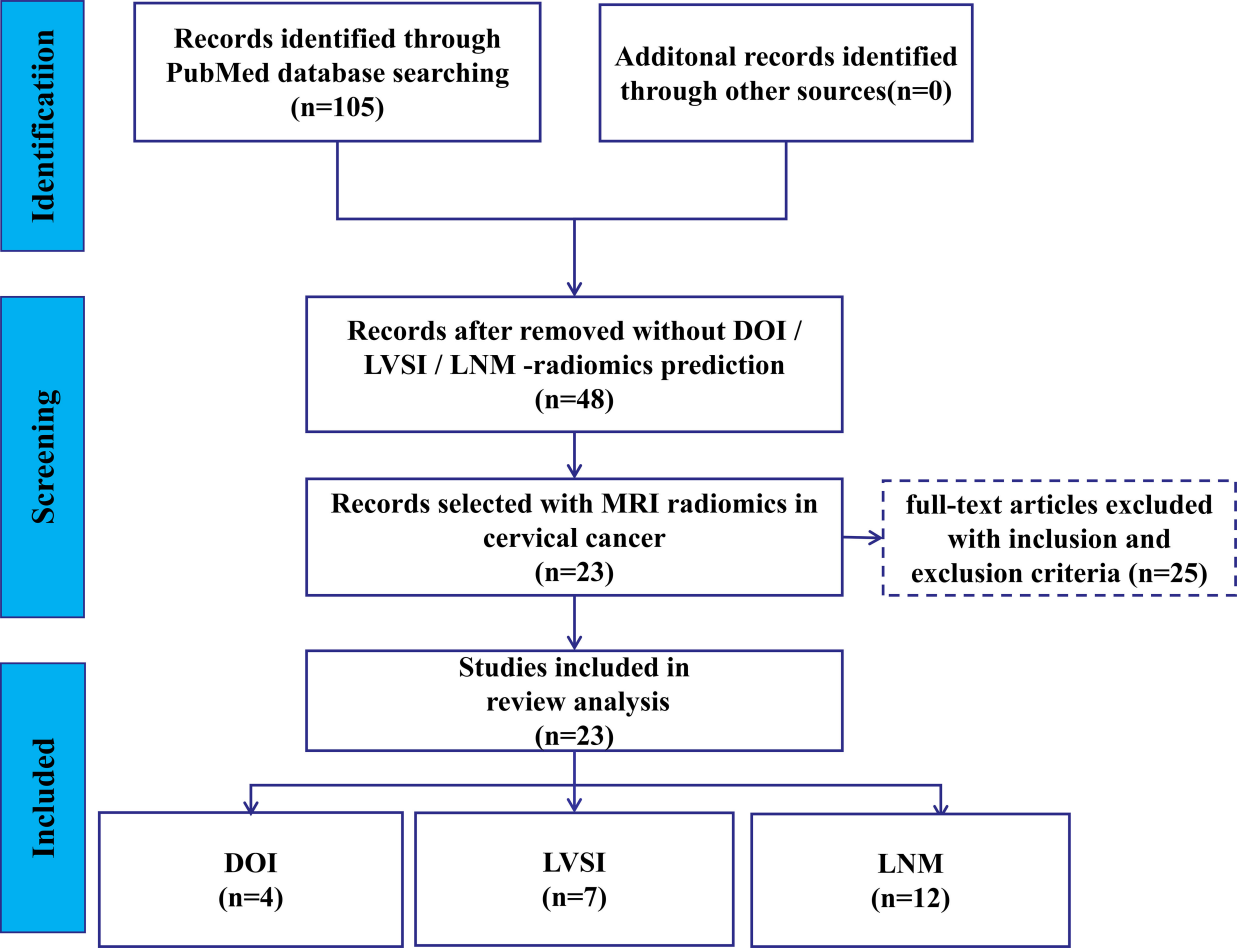
Flow chart of this study.

The flowchart depicting search scheme was presented in **Figure 1**. Among the initial 105 studies retrieved, 57 studies were excluded due to their irrelevance to the current review. A further 12 studies were eliminated as they failed to provide data on TP, TN, FP, and FN, leaving 48 studies for consideration. Out of the remaining 36 studies that met the requirements, the following studies were further excluded: 2 MRI studies based on deep learning algorithms (24, 25); 3 studies based on PET/CT images (26–28), one study based on CT images (29), and 2 studies based on US images (30, 31); 3 studies on predicting the LNM status of CC primary lesions based on lymph node imaging (32–34). Ultimately, 25 studies were selected for analysis. Among these, 1 study was utilized to predict both DOI and LNM (35), while another study was utilized to predict both LVSI and LNM (36). Consequently, 5, 8, and 14 MRI R studies were included in the meta-analysis of DOI (14, 35, 37–39), LVSI (36, 40–46), and LNM (16, 17, 35, 36, 47–56), respectively.

### 3.2 Study quality assessment

All the included studies exhibited a retrospective design, and only five of them utilized multicenter data. According to the first author’s affiliation, 24 studies were conducted in China, while the remaining one were from Japan. A total of 24 studies provided details on cohort selection criteria and image protocols. Regarding image acquisition, 19 studies were relied a single scanner (4 GE, 6 Philips, and 9 Siemens), 3 studies were utilized two scanners (GE, Philips, and Siemens), and 3 studies remained unclear about the scanner used. For image segmentation, 8 studies employed a single sequence segmentation, 10 studies used two sequence, and 7 studies utilized three or more sequence. 23 studies adopted multi person segmentation, while 13 studies focused on the robustness of intrahepatic cholangiocarcinoma (ICC) validation features. The primary segmentation software included ITK-SNAP and 3D slicer, and 24 studies performed manual segmentation. Prior to feature extraction, 15 studies underwent image normalization processing. Feature extraction was mainly based on the Pyradiomics software package, with 19 study extracting over 1000 features, significantly exceeding the cohort population. All studies employed multi-step feature selection strategies, among which 10 sutides utilized logistic regression (LR), 7 sutides utilized support vector machine (SVM), and 6 sutides utilized least absolute shrinkage and selection operator (LASSO) algorithm to construct models. In terms of model evaluation, 13 studies opted for cross-validation to obtain optimal results, while 7 studies compared model performance using the Delong test. 24 studies conducted internal or external validation of the models. 14 studies performed multi-factor analysis on clinical or R features, and 21 studies constructed R-C models. 15 studies underwent model calibration, and 12 studies reported decision curve results.

The methodological quality of the studies was rigorously evaluated by RQS and QUADAS-2 tools. The results indicated varied methodological quality across the studies, with RQS scores for DOI, LVSI, and LNM ranging from 5–17, 2–17, and 2–17, respectively. Consequently, two studies with RQS scores below 10 were deemed unfit for inclusion and were excluded from further analysis (35, 36). The QUADAS-2 evaluation revealed that in terms of patient selection, 24 studies displayed a low risk of bias, while only 1 study exhibited a higher risk. Similarly, in the index test category, 24 studies demonstrated a low risk of bias, with 1 study identified as having a higher risk. The reference standard test showed a consistently low risk in 25 studies. However, regarding the flow and timing of the studies, the risk of bias remained unclear in all 25 studies.

**Figure 2:**
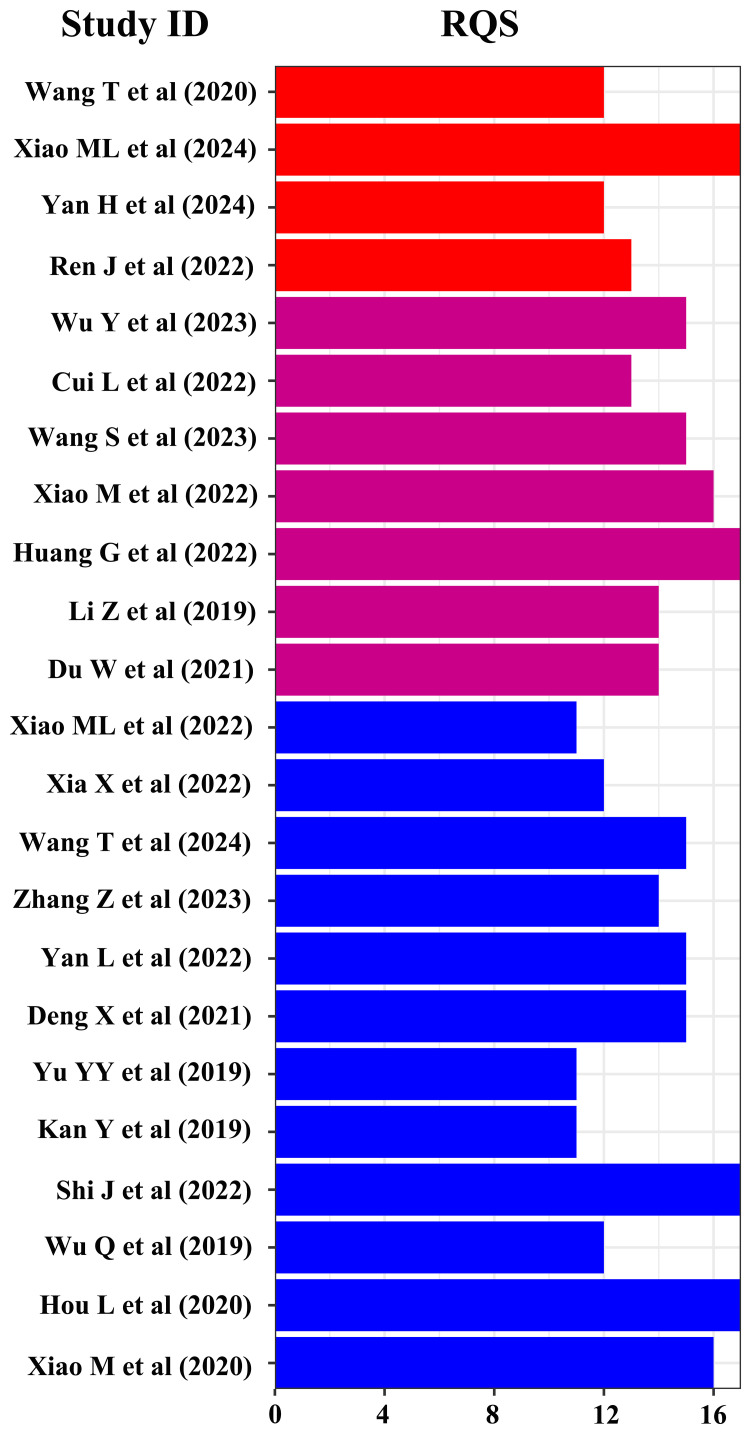
RQS evaluation of included studies.

**Figure 3:**
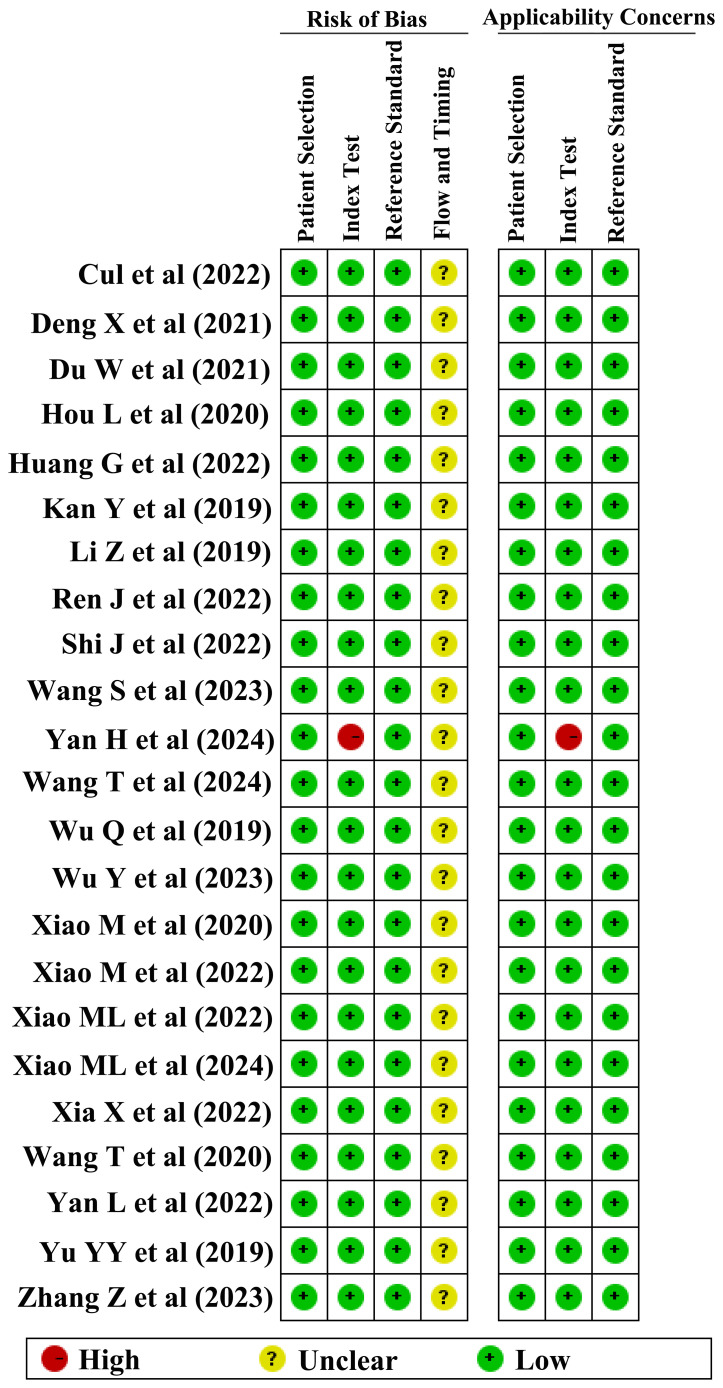
QUADAS-2 evaluation of included studies.

Finally, 4, 7, and 12 studies were included in the meta-analysis for DOI, LVSI, and LNM, respectively (**Table 1**). The RQS scores in these studies ranges from 12–17, 13–17, and 11–17, with an average scores of 13.5, 14.9, and 13.8, respectively (**Figure 2**). Notably, only one study in the patient selection test and one in the index test category were classified as high-risk (**Figure 3**).

**Table 1:** Basic characteristics of included studies.

Study ID	Year	Country	Type	Center	Scanner	Sequence	Segmentation software	Segmentation method	Extraction software	Feature number	Algorithm
Ren J et al	2022	China	Retrospective	Single	GE	T2WI	InferScholar center	Manual	InferScholar center	1454	LR
Wang T et al	2020	China	Retrospective	Single	Philips	T2WI, DWI	3D slicer	Manual	MATLAB	1046	SVM
Yan H et al	2024	China	Retrospective	Single	Unclear	T2WI, CE-T1WI	3D slicer	Semi-automatic	PyRadiomics	2632	LightGBM
Xiao ML et al	2024	China	Retrospective	Multiple	Siemens, GE	T1WI, DWI, CE-T1WI	ITK-SNAP	Manual	Unclear	2364	LASSO
Li Z et al	2019	China	Retrospective	Single	GE	CE-T1WI	ITK-SNAP	Manual	PyRadiomics	1392	LR
Du W et al	2021	China	Retrospective	Single	Philips	T2WI	3D slicer	Manual	PyRadiomics	1682	SVM
Huang G et al	2022	China	Retrospective	Single	Siemens	sFOV-T2WI, ADC, T2WI, FS-T2WI	ITK-SNAP	Manual	PyRadiomics	1037	LR
Xiao M et al	2022	China	Retrospective	Single	Siemens	T1WI, FS-T2WI, DWI, ADC, CE-T1WI	MITK	Manual	PyRadiomics	3940	LASSO
Wang S et al	2023	China	Retrospective	Multiple	Siemens	CE-TIWI	ITK-SNAP	Manual	PyRadiomics	1016	SVM
Cui L et ak	2022	China	Retrospective	Single	Siemens	T2WI, CE-TIWI	ITK-SNAP	Manual	PyRadiomics	2990	LR
Wu Y et al	2023	China	Retrospective	Multiple	Unclear	T2WI, CE-TIWI	ITK-SNAP	Manual	PyRadiomics	1132	LR
Wu Q et al	2019	China	Retrospective	Single	GE, Siemens	T2WI, DWI	ITK-SNAP	Manual	PyRadiomics	1299	SVM
Kan Y et al	2019	China	Retrospective	Single	Siemens	T2WI, CE-TIWI	ITK-SNAP	Manual	MATLAB	465	SVM
Yu YY et al	2019	China	Retrospective	Single	Siemens	ADC	Omni-Kinetics	Manual	Omni-Kinetics	66	LR
Xiao M et al	2020	China	Retrospective	Single	Siemens	T1WI, FS-T2WI, CE-T1WI, DWI, ADC	MITK	Manual	PyRadiomics	3490	LASSO
Hou L et al	2020	China	Retrospective	Multiple	Unclear	T2WI, ADC, CE-TIWI	ITK-SNAP	Manual	PyRadiomics	3390	LR
Deng X et al	2021	China	Retrospective	Single	Philips	T2WI, CE-TIWI	Philips radiomics tool	Manual	Philips radiomics tool	3386	Unclear
Shi J et al	2022	China	Retrospective	Multiple	Siemens	T2WI, CE-TIWI	ITK-SNAP	Manual	PyRadiomics	1967	LR
Xiao ML et al	2022	China	Retrospective	Single	Siemens	T2WI, DWI	MITK	Manual	PyRadiomics	272	SVM
Xia X et al	2022	China	Retrospective	Single	Philips	T2WI	3D slicer	Manual	PyRadiomics	1688	SVM
Yan L et al	2022	China	Retrospective	Single	GE, Philips	FS-T2WI	ITK-SNAP	Manual	MATLAB	8715	LASSO
Zhang Z et al	2023	China	Retrospective	Single	Philips	T2WI, DWI	3D slicer	Manual	PyRadiomics	1014	LASSO
Wang T et al	2024	China	Retrospective	Single	Philips	T2WI, SPAIR-T2WI, ADC	3D slicer	Manual	3D slicer	851	LASSO

### 3.3 Preoperative MRI radiomics models for predicting DOI in CC

**Figure 4:**
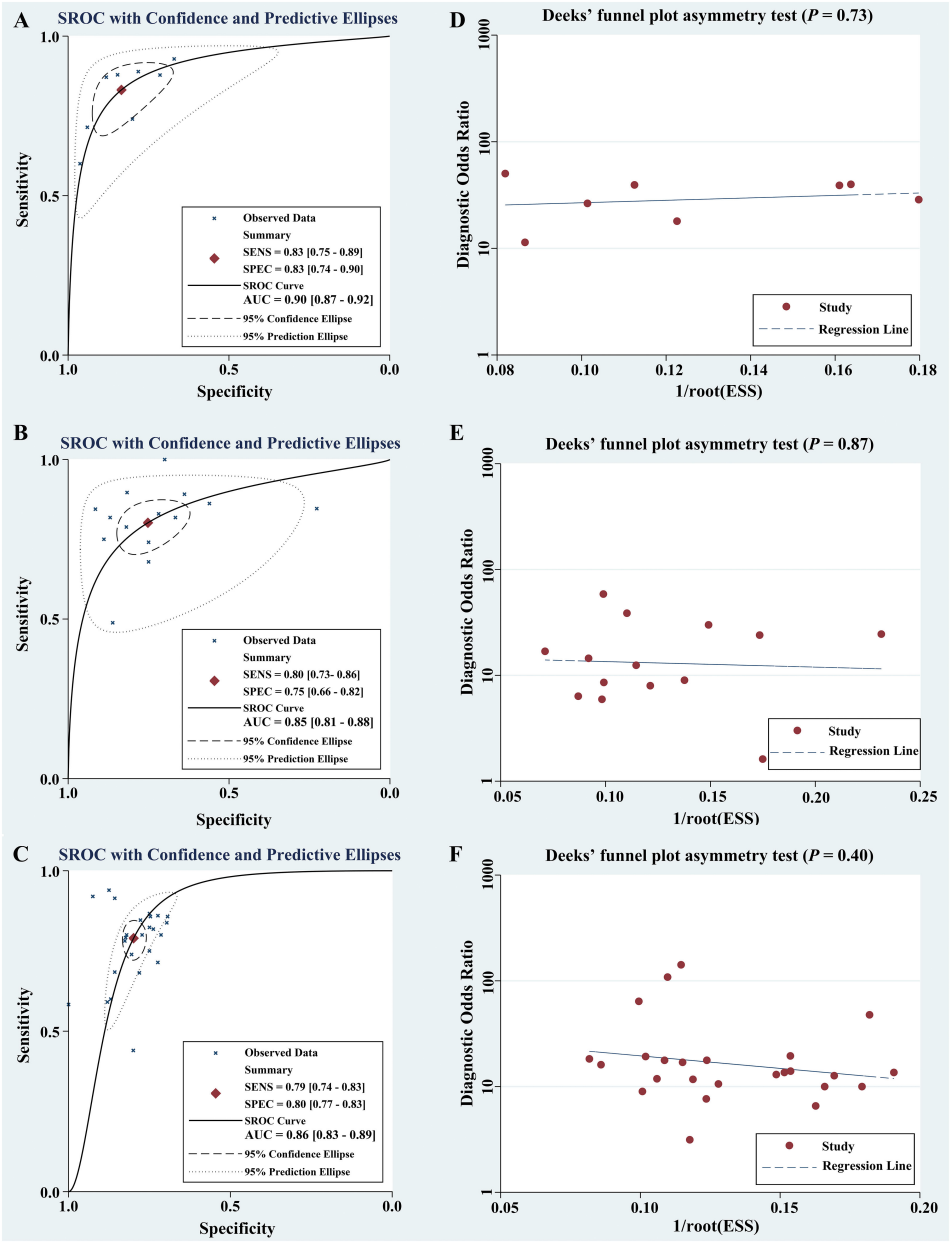
Comprehensive potential evaluation in CC based on preoperative MRI radiomics. Through sROC curve analysis, the meta-analyses based on DOI **(A)**, LVSI **(B)**, and LNM **(C)** showed overall AUC of 0.90, 0.85, and 0.86, respectively. The presence of publication bias was detected by Deek’s funnel plots, and the results showed no publication bias [DOI, P=0.73, **(D)**;LVSI, P=0.87, **(E)**; LNM, P=0.40, **(F)**].

**Table 2** summarized the basic characteristics of 4 studies aimed at predicting DOI in CC. These studies encompassed a total of 8 cohorts, comprising 839 CC patients, including 305 patients with DOI and 534 patients without DOI. The AUC, SENC, and SPEC of all cohorts ranged from 0.83–0.95, 0.60–0.93, and 0.67–0.96, respectively. Furthermore, 3 studies incorporated both R and C models, as well as their R-C models (**Table 3**). The results indicated that the overall SENC and SPEC of R, C, R-C models were 0.83 (95% confidence interval [CI], 0.75–0.89) and 0.83 (95% CI, 0.74–0.90); 0.79 (95% CI, 0.70–0.86) and 0.72 (95% CI, 0.65–0.78); 0.91 (95% CI, 0.86–0.94) and 0.84 (95% CI, 0.73–0.91), respectively. Notably, significant heterogeneity was observed in both overall SENC (I^2^ = 54.74%, *P*=0.03) and SPEC (I^2^ = 82.34%, *P <*0.01) of R models. Through sROC curve analysis, the overall AUC of R, C, R-C models were 0.90, 0.82, 0.94, respectively, indicating superior evaluation performance (**Figure 4A**). Deek’s funnel plots were utilized to detect publication bias in the R models, and the results indicated the absence of such bias (*t*=0.36, *P*=0.73) (**Figure 4B**).

**Table 2:** Preoperative MRI Radiomics Models for Predicting DOI in CC.

Model	Study ID	N (T/V)	AUC (T/V)	SENC (T/V)	SPEC (T/V)	TP (T/V)	FP (T/V)	FN (T/V)	TN (T/V)
R	Ren J et al	-/46	-/0.88	-/0.88	-/0.85	-/29	-/2	-/4	-/11
	Yan H et al	160/69	0.95/0.88	0.87/0.88	0.88/0.71	88/36	7/8	13/5	52/20
	Xiao ML et al	150/213/64	0.84/0.83/0.91	0.74/0.93/0.89	0.8/0.67/0.78	37/26/8	20/61/12	13/2/1	80/124/43
	Wang T et al	95/42	0.95/0.92	0.71/0.60	0.94/0.96	20/9	4/1	8/6	63/26
C	Ren J et al	-/46	-/0.84	-/0.70	-/0.77	-/23	-/3	-/10	-/10
	Yan H et al	160/69	0.77/0.77	0.68/0.76	0.85/0.71	69/31	9/8	32/10	50/20
	Xiao ML et a	150/213/64	0.79/0.86/0.73	0.86/0.93/0.78	0.66/0.69/0.64	43/26/7	34/57/20	7/2/2	66/128/35
R-C	Ren J et al	-/46	-/0.89	-/0.88	-/0.85	-/29	-/2	-/4	-/11
	Yan H et al	160/69	0.97/0.91	0.90/0.83	0.93/0.86	91/34	4/4	10/7	55/24
	Xiao ML et al	150/213/64	0.87/0.89/0.97	0.92/1.00/0.89	0.68/0.71/0.93	46/28/8	32/54/4	4/0/1	68/131/51

**Table 3:** Clinical factors for constructing combined models.

Study ID	Target	Clinical factors
Ren J et al	DOI	Maximal tumor diameter on MRI
Yan H et al		FIGO stage, squamous cell carcinoma antigen
Xiao ML et al		FIGO stage, cancer antigen 125, maximal tumor diameter on MRI, lymph node metastasis on MRI, disruption of cervical stromal ring on MRI
Du W et al	LVSI	Stromal invasion depth, maximal tumor diameter on MRI, FIGO stage
Li Z et al		Red blood cell
Huang G et al		Hemoglobin, squamous cell carcinoma antigen
Xiao M et al		Age, tumor size, lymph node status on MRI
Wu Y et al		Age, low signal ring of cervical stroma, lymphatic metastasis
Xiao M et al	LNM	lymph node status on MRI, FIGO stage
Hou L et al		lymph node status on MRI
Wu Q et al		Lymph node status on MRI, maximal tumor diameter on MRI, FIGO stages
Shi J et al		Lymph node status on MRI, maximal tumor diameter on MRI
Deng X et al		FIGO stage
Wang T et al		Diferentiation level, FIGO stage
Xia X et al		Stromal invasion depth, FIGO stage, lymph node status on MRI
Xiao ML et al		Lymph node status on MRI

A subgroup analysis of the R models based on cohort type revealed no significant difference in the overall AUC between the training and validation cohorts (AUC, 0.90 vs 0.91). However, the overall SENC of the validation cohorts was slightly higher, while the overall SPEC was slightly lower (**Table 4**).

**Table 4:** Subgroup analysis of MRI radiomics for predicting DOI, LVSI, and LNM in patients with CC.

Target	Co-variate	Subgroup	SENC	SPEC	AUC
DOI	Cohort	Training	0.81 (0.74-0.86)	0.86 (0.81-0.90)	0.90
		Validaton	0.86 (0.78-0.91)	0.73 (0.67-0.78)	0.91
LVSI	Cohort	Training	0.76 (0.71-0.80)	0.77 (0.73-0.81)	0.86
		Validaton	0.80 (0.73-0.86)	0.70 (0.63-0.76)	0.85
	Center	Single	0.76 (0.71-0.80)	0.75 (0.71-0.79)	0.85
		Multiple	0.80 (0.74-0.85)	0.75 (0.70-0.81)	0.79
	Scanner	Siemens	0.74 (0.69-0.78)	0.78 (0.74-0.82)	0.84
		Non-Siemens	0.85 (0.79-0.90)	0.70 (0.64-0.76)	0.91
	Sequence	Single	0.80 (0.75-0.85)	0.75 (0.69-0.80)	0.87
		Two	0.72 (0.64-0.80)	0.75 (0.68-0.80)	0.83
		Three or over three	0.76 (0.69-0.82)	0.77 (0.70-0.83)	0.82
	Algorithm	LR	0.78 (0.72-0.84)	0.70 (0.65-0.75)	0.84
		SVM	0.79 (0.73-0.84)	0.83 (0.78-0.88)	0.81
	Cross validation	With	0.86 (0.80-0.91)	0.79 (0.74-0.83)	0.92
		Without	0.74 (0.69-0.78)	0.73 (0.68-0.77)	0.81
LNM	Cohort	Training	0.82 (0.78-0.86)	0.80 (0.78-0.83)	0.87
		Validaton	0.72 (0.66-0.78)	0.78 (0.74-0.82)	0.84
	Center	Single	0.79 (0.75-0.82)	0.80 (0.78-0.83)	0.87
		Multiple	0.79 (0.70-0.86)	0.77 (0.71-0.82)	0.86
	Scanner	Philips	0.78 (0.71-0.84)	0.82 (0.78-0.85)	0.87
		Siemens	0.78 (0.73-0.83)	0.78 (0.74-0.81)	0.85
	Sequence	Single	0.77 (0.69-0.83)	0.81 (0.77-0.85)	0.87
		Two	0.79 (0.75-0.84)	0.81 (0.77-0.84)	0.87
		Three or over three	0.79 (0.72-0.85)	0.76 (0.72-0.81)	0.85
	Algorithm	LASSO	0.74 (0.68-0.80)	0.80 (0.76-0.83)	0.86
		LR	0.81 (0.74-0.87)	0.77 (0.72-0.81)	0.87
		SVM	0.80 (0.73-0.86)	0.81 (0.77-0.85)	0.87
	Cross validation	With	0.80 (0.74-0.84)	0.79 (0.75-0.82)	0.85
		Without	0.78 (0.73-0.82)	0.80 (0.77-0.83)	0.87

### 3.4 Preoperative MRI radiomics models for predicting LVSI in CC

**Table 5** summarized the basic characteristics of 7 studies aimed at predicting LVSI in CC. These studies encompassed a total of 14 cohorts, comprising 1243 CC patients, including 577 patients with LVSI and 666 patients without LVSI. The AUC, SENC, and SPEC of all cohorts ranged from 0.63–0.94, 0.49–1.00, and 0.23–0.91, respectively. Furthermore, 3 studies focused solely on C models, while 5 studies integrated R-C models into their analysis (**Table 3**). The results indicated that the overall SENC and SPEC of R, C, R-C models were 0.80 (95% CI, 0.73-0.86) and 0.75 (95% CI, 0.66–0.82); 0.52 (95% CI, 0.42–0.62) and 0.92 (95% CI, 0.81–0.97); 0.84 (95% CI, 0.79–0.87) and 0.82 (95% CI, 0.78–0.85), respectively. Notably, significant heterogeneity was observed in both overall SENC (I^2^ = 65.90%, *P*<0.01) and SPEC (I^2^ = 81.10%, *P*<0.01) of R models. Through sROC curve analysis, the overall AUC of R, C, R-C models were 0.85, 0.67, 0.89, respectively, indicating superior evaluation performance (**Figure 4C**). The existence of publication bias of R models was detected by Deek’s funnel plots were utilized to detect publication bias in the R models, and the results indicated the absence of such bias (*t*=-0.16, *P*=0.87) (**Figure 4D**).

**Table 5:** Preoperative MRI Radiomics Models for Predicting LVSI in CC.

Model	Study ID	N (T/V)	AUC (T/V)	SENC (T/V)	SPEC (T/V)	TP (T/V)	FP (T/V)	FN (T/V)	TN (T/V)
R	Du W et al	104/45	0.93/0.91	0.84/0.81	0.91/0.86	38/18	5/3	7/4	54/20
	Li Z et al	70/35	0.71/0.63	0.85/0.82	0.55/0.23	25/11	18/17	4/2	23/5
	Huang G et al	100/25	0.92/0.94	0.90/1.00	0.82/0.70	26/5	13/6	3/0	58/14
	Xiao M et al	154/79	0.76/0.81	0.68/0.84	0.75/0.71	72/39	12/9	34/8	36/23
	Wang S et al	198/102	0.87/0.78	0.79/0.74	0.82/0.75	82/40	17/12	22/14	77/36
	Cui L et al	108/55	0.71/0.76	0.49/0.82	0.86/0.67	21/18	9/11	22/4	56/22
	Wu Y et al	129/39	0.84/0.78	0.89/0.75	0.64/0.89	41/9	30/3	5/3	53/24
C	Du W et al	104/45	0.79/0.71	0.42/0.43	0.95/0.95	19/9	3/1	26/13	56/22
	Huang G et al	100/-	0.71/-	0.66/-	0.66/-	19 /-	24 /-	10/-	47/-
	Wu Y et al	129/39	0.81/0.69	0.54/0.58	0.96/0.92	25/7	3/2	21/5	80/25
R-C	Du W et al	104/45	0.94/0.92	0.85/0.84	0.93/0.85	38/18	4/3	7/4	55/20
	Li Z et al	70/35	0.75/0.73	0.83/0.69	0.76/0.77	24/9	10/5	5/4	31/17
	Huang G et al	100/-	0.92/-	0.90/-	0.82/-	26/-	13/-	3/-	58/-
	Xiao M et al	154/79	0.78/0.82	0.90/0.80	0.58/0.77	95/38	20/7	11/9	28/25
	Wu Y et al	129/39	0.88/0.83	0.74/0.83	0.92/0.81	34/10	7/5	12/2	76/22

A subgroup analysis of R models was conducted, considering factors such as cohort type, number of centers, scanner type, sequence number, model algorithm, and cross-validation. The findings revealed that no significant difference in the overall AUC between the training and validation cohorts (AUC, 0.86 vs 0.85). Interestingly, studies involving multiple centers demonstrated a lower overall AUC than those from a single center, yet they exhibited higher SENC. When analyzing scanner types, a single Siemens scanner yielded an AUC of 0.84, indicating moderate performance. The sequence number and algorithm types did not seem to significantly impact performance. Notably, cross-validated studies exhibited superior performance, with an AUC of 0.92 compared to 0.81 (**Table 4**).

### 3.5 Preoperative MRI radiomics models for predicting LNM in CC

**Table 6** summarized the basic characteristics of 12 studies aimed at predicting LNM in CC. These studies encompassed a total of 25 cohorts, comprising 2004 CC patients, including 631patients with LNM and 1373 patients without LNM. The AUC, SENC, and SPEC of all cohorts ranged from 0.70–0.98, 0.44–0.94, and 0.69–1.00, respectively. Furthermore, 7 studies focused solely on C models, while 8 studies integrated R-C models into their analysis (**Table 3**). The results indicated that the overall SENC and SPEC of R, C, R-C models were 0.79 (95% CI, 0.74-0.83) and 0.80 (95% CI, 0.77-0.83); 0.69 (95% CI, 0.62-0.74) and 0.79 (95% CI, 0.70-0.86); 0.84 (95% CI, 0.80-0.88) and 0.84 (95% CI, 0.79-0.89), respectively. Notably, significant heterogeneity was observed in both overall SENC (I^2^ = 50.67%, *P*<0.01) and SPEC (I^2^ = 36.24%, *P*=0.04) of R models. Through sROC curve analysis, the overall AUC of R, C, R-C models were 0.86, 0.76, 0.91, respectively, indicating superior evaluation performance (**Figure 4E**, **Table 7**). Deek’s funnel plots were utilized to detect publication bias in the R models, and the results indicated the absence of such bias (*t*=-0.87, *P*=0.40) (**Figure 4F**).

**Table 6:** Preoperative MRI Radiomics Models for Predicting LNM in CC.

Model	Study ID	N (T/V)	AUC (T/V)	SENC (T/V)	SPEC (T/V)	TP (T/V)	FP (T/V)	FN (T/V)	TN (T/V)
R	Xiao M et al	155/78	0.86/0.88	0.86/0.78	0.72/0.83	43/25	29/8	7/7	76/38
	Hou L et al	115/53	0.86/0.83	0.86/0.82	0.75/0.74	24/9	22/11	4/2	65/31
	Wu Q et al	126/63	0.98/0.79	0.91/0.86	0.86/0.69	32/12	13/15	3/2	78/34
	Shi J et al	93/47/29	0.83/0.85/0.70	0.83/0.68/0.58	0.69/0.85/1.00	31/13/7	17/4/0	6/6/5	39/24/17
	Kan Y et al	100/43	0.75/0.75	0.75/0.71	0.75/0.72	33/10	14/8	11/4	42/21
	Yu YY et al	102/51	0.86/0.87	0.85/0.87	0.78/0.75	33/13	14/9	6/2	49/27
	Deng X et al	89/45	0.94/0.87	0.94/0.82	0.88/0.75	31/14	7/7	2/3	49/21
	Yan L et al	100/90	0.79/0.73	0.74/0.44	0.81/0.80	17/11	15/13	6/14	62/52
	Zhang Z et al	172/75	0.87/0.85	0.80/0.78	0.82/0.83	44/19	21/9	11/5	96/42
	Wang T et al	86/38	0.84/0.83	0.68/0.60	0.78/0.87	15/9	14/3	7/6	50/20
	Xia X et al	105/45	0.98/0.85	0.92/0.83	0.92/0.71	23/8	6/10	2/2	74/25
	Xiao ML et al	72/32	0.78/0.82	0.59/0.80	0.88/0.77	13/8	6/5	9/2	44/17
C	Wu Q et al	126/63	0.73/0.72	0.52/0.57	0.97/0.94	18/8	3/3	17/6	88/46
	Kan Y et al	100/43	-/-	0.86/0.71	0.48/0.38	38/10	29/18	6/4	27/11
	Deng X et al	89/45	0.78/0.73	0.70/0.71	0.80/0.64	23/12	11/10	10/5	45/18
	Yan L et al	100/90	0.74/0.69	0.65/0.56	0.82/0.82	15/14	14/12	8/11	63/53
	Shi J et al	93/47/29	0.81/0.82/0.79	0.75/0.73/0.75	0.78/0.82/0.76	28/14/9	12/5/4	9/5/3	44/23/13
	Xia X et al	105/45	0.93/0.84	0.70/0.66	0.92/0.83	18/7	6/6	7/3	74/29
	Xiao ML et al	72/32	0.62/0.60	0.50/0.60	0.74/0.59	11/6	13/9	11/4	37/13
R-C	Wu Q et al	126/63	0.90/0.85	0.94/1.00	0.85/0.69	33/14	14/15	2/0	77/34
	Xiao M et al	155/78	0.88/0.89	0.78/0.84	0.86/0.76	39/27	15/11	11/5	90/35
	Hou L et al	115/53	0.87/0.86	0.93/0.82	0.70/0.74	26/9	26/11	2/2	61/31
	Deng X et al	89/45	0.95/0.88	0.88/0.82	0.89/0.82	29/14	6/5	4/3	50/23
	Xia X et al	105/45	0.99/0.92	0.92/0.86	1.00/0.89	23/9	0/4	2/1	80/31
	Shi J et al	93/47/29	0.89/0.76/0.80	0.86/0.63/0.75	0.82/0.96/0.88	32/12/9	10/1/2	5/7/3	46/27/15
	Wang T et al	86/38	0.92/0.82	0.82/0.67	0.86/0.78	18/10	9/5	4/5	55/18
	Xiao ML et al	72/32	0.79/0.79	0.71/0.90	0.84/0.64	16/9	8/8	6/1	42/14

**Table 7:** Performance of preoperative MRI radiomics this review.

Target	Model	SENC	SPEC	AUC
DOI	R	0.83 (0.75-0.89)	0.83 (0.74-0.90)	0.90
	C	0.79 (0.70-0.86)	0.72 (0.65-0.78)	0.82
	R-C	0.91 (0.86-0.94)	0.84 (0.73-0.91)	0.94
LVSI	R	0.80 (0.73-0.86)	0.75 (0.66-0.82)	0.85
	C	0.52 (0.42-0.62)	0.92 (0.81-0.97)	0.67
	R-C	0.84 (0.79-0.87)	0.82 (0.78-0.85)	0.89
LNM	R	0.79 (0.74-0.83)	0.80 (0.77-0.83)	0.86
	C	0.69 (0.62-0.74)	0.79 (0.70-0.86)	0.76
	R-C	0.84 (0.80-0.88)	0.84 (0.79-0.89)	0.91

A thorough subgroup analysis was performed on R models, encompassing factors such as cohort type, center number, scanner type, sequence number, model algorithm, and cross-validation. The findings indicate a marginal difference in performance between the training and validation cohorts, with AUC values of 0.87 and 0.84, respectively. The number of centers did not significantly influence performance, with AUC values 0.87 and 0.86. Similarly, Philips and Siemens scanners displayed comparable performance, with AUCs of 0.87 and 0.85, respectively. Additionally, the sequence number, algorithm types, and cross-validation appeared to have minimal impact on the models’ overall performance (**Table 4**).

### 3.6 Meta-analysis investigation of preoperative radiomics in uterine cancer

The meta-analysis investigating preoperative MRI R models for uterine cancer was summarized in **Table 8**. To our knowledge, no previous meta-analysis had been found to predict DOI and LVSI in CC. 2 meta-analyses were found for LNM prediction in CC (57, 58), reporting overall SENC and SPEC of 0.80 and 0.76; 0.84 and 0.73, respectively. Additionally, 2 meta-analyses were simultaneously found for DOI, LVSI and LNM prediction in EC (18, 59). 1 meta-analyses were solely found for LVSI prediction in EC (60).

**Table 8:** Meta analysis investigation of preoperative radiomics in uterine cancer.

Study ID	Year	Cancer	Target	Study number	AUC	SENC	SEPC
Li L et al	2022	CC	LNM	12	0.83	0.80 (0.72-0.87)	0.76 (0.72-0.80)
Ren J et al	2022	CC	LNM	8	0.86	0.84 (0.73-0.91)	0.73 (0.62-0.81)
He J et al	2024	EC	LNM	T: 11 V: 8	- -	0.76 (0.69-0.82) 0.87 (0.74-0.93)	0.83 (0.75-0.88) 0.80 (0.69-0.87)
			LVSI	T: 9 V: 8	- -	0.85 (0.76-0.91) 0.76 (0.64-0.84)	0.76 (0.66-0.84) 0.76 (0.65-0.84)
			DOI	T: 13 V: 10	- -	0.80 (0.74-0.84) 0.75 (0.68-0.82)	0.81 (0.76-0.86) 0.81 (0.73-0.88)
Di Donato V et al	2023	EC	LNM	4		0.83 (0.63-0.93)	0.74 (0.60-0.84)
			LVSI	5	–	0.66 (0.56-0.74)	0.75 (0.60-0.86)
			DOI	4	–	0.74 (0.61-0.84)	0.82 (0.74-0.87)
Meng X et al	2023	EC	LVSI	9	0.82	0.73	0.77

## 4 Discussion

### 4.1 Principal findings

In comparison to traditional MRI visual imaging, R base on MRI images harnesses a broader range of image information to assess preoperative biological characteristics of uterine cancer, encompassing DOI, LVSI, and LNM. To our knowledge, this systematic review and meta-analysis was the first comprehensive analysis of preoperative R, C, and R-C models in predicting DOI, LVSI, and LNM, aiming to elucidate the potential of R in preoperative evaluation of CC biological characteristics. The meta-analysis results demonstrated satisfactory diagnostic accuracy. For the meta-analysis of DOI, the results based on R, C, and R-C showed overall SENS, SPEC, and AUC of 0.83, 0.83, and 0.90; 0.79, 0.72, and 0.82; 0.91, 0.84, and 0.94, respectively. In the meta-analysis of LVSI, the corresponding values for R, C, and R-C models were 0.80, 0.75, and 0.85; 0.52, 0.92, and 0.67; 0.84, 0.82, and 0.89, respectively. Finally, for LNM, the corresponding values for R, C, and R-C models were 0.79, 0.80, and 0.86; 0.69, 0.79, and 0.76; 0.84, 0.84, and 0.91, respectively. Compared to the R models, the R-C models exhibited a marginal improvement in performance, while significantly outperforming C models. Although R had shown great potential in predicting CC biological characteristics, it also exhibited high heterogeneity in meta-analyses, with RQS scores indicating a higher methodological quality risk, similar to other R meta-analyses. This highlights urgent need for the development of the more reliable methodological quality standardization process in R.

R, analogous to gene sequencing, quantifies the high-throughput tumor information embedded in medical images, thereby enhancing the utilization of image data in clinical decision-making. This approach has the potential to address clinical challenges that traditional qualitative imaging diagnosis cannot, approaching the explanatory power of genomics in diseases. The application of MRI-based R has been extensively explored in elucidating the biological characteristics of uterine cancer, with over a hundred publications, particularly in EC. In a meta-analysis encompassing 33 preoperative studies, R demonstrated promising SENC and SPEC in predicting DOI, LVSI, and LNM were 0.75 and 0.81; 0.76 and 0.76; 0.87 and 0.80, respectively (56); a separate meta-analysis based on five studies revealed similar trends, with SENC and SPEC values of 0.74 and 0.82; 0.66 and 0.75; 0.83 and 0.74, respectively (18). However, in the context of CC, meta-analytic reports in 2022 appeared to be limited to predictions of LNM. For example, scholars such as Li L conducted a meta-analysis based on 12 studies, revealing a predictive performance of 0.83 for preoperative LNM (57); Ren J and other scholars conducted a meta-analysis based on 8 studies, reported a predictive performance of 0.86 for preoperative LNM using image-based R (58). Notably, there was a significant overlap between the studies included in these two meta-analyses, suggesting a quantitative expansion rather than qualitative advancement. Therefore, the predictive potential of MRI-R for DOI and LVSI in CC remains an area requiring further exploration and elucidation.

Despite its promise, R encounters notable challenges in the standardization and assessment of methodological quality. Some scholars had proposed that R need to establish rigorous evaluation and reporting standards, namely RQS. However, in practice, the RQS scores in various studies tend to be relatively low. For example, an analysis encompassing 33 studies on EC revealed an average RQS of 7 points, ranging from 5 to 12 (59). In a CC meta-analysis of 8 studies, the mean RQS was 13.5, spanning from 6 to 16 points (58). The average RQS for DOI, LVSI, and LNM in this review were 13.5, 14.9, and 13.8, respectively, aligning with findings from other retrospective studies. According to the results of the QUADAS-2 tool, only one study exhibited a high risk in the index test, while the remainder presented low risks in patient selection and reference standard. However, both the flow and timing tests showed unclear risk levels, a trend consistent with other meta-analyses (61). The findings of this review and other evaluations suggest that the RQS does not fully capture the quality of study design in its current application, necessitating a more comprehensive and standardized evaluation process.

In this review, for the first time, the potential of R in predicting the biological characteristics of CC was comprehensively analyzed based on R, C, and R-C models. To ensure the reliability of the conclusions, methodological quality assessment was utilized to exclude low-quality studies. Although the number of studies included was small, the results showed satisfaction. In the comprehensive analysis of DOI, LVSI, and LNM, the R models performed better than the C model, slightly lower than the R-C models. Both R and R-C models exhibit moderate to superior performance. However, the comprehensive analysis of R revealed a high degree of heterogeneity. Drawing parallels from other meta-analyses, this heterogeneity is closely associated with factors such as cohort types, number of centers, scanner types, image protocols, number of sequences, algorithm variations, and the implementation of cross-validation. To further explore the sources of heterogeneity, this meta-analysis also conducted subgroup analysis on the sources of heterogeneity, and the results showed that in the DOI analysis, there seemed to be no significant difference in overall performance between cohorts. The SENC of the validation cohort was higher, but the SPEC was lower than that of the training cohort. In the analysis of LVSI, the validation cohorts seemed to outperform the training cohort, with higher SENC. The number of centers and algorithm types did not significantly influence performance, while cross-validation appeared to enhance predictive capabilities. In the LNM analysis, the performance between cohorts was comparable, with the training cohort demonstrating higher SENC and the validation cohorts exhibiting higher SPEC. The number of centers, number of sequences, algorithm types, and cross-validation did not appear to have a significant impact on predictive performance. This subgroup analysis, based on the overall AUC, did not significantly affect the analysis of DOI and LNM, while the analysis of LVSI seemed to be influenced by cohort type and cross validation.

### 4.2 Practical implications

The preoperative biological behavior assessment of CC patients, such as DOI, LVSI, and LNM, was beneficial for clinical decision-making. In practice, traditional MRI imaging mainly relied on visual information to evaluate the biological behavior of CC patients. However, the limitations of visual information, differences in equipment, and the long-term cultivation of visual experience by physicians made it difficult to meet the needs of precision medicine in terms of evaluation results. In addition, clinical information also could not effectively evaluate the biological behavior of CC patients. In summary, more effective and timely methods are needed for preoperative biological evaluation of CC patients. The R model based on AI broke through the limitations of the naked eye and greatly improved the utilization of MRI image information. Its prediction of DOI, LVSI, and LNM in CC patients was satisfactory, surpassing the level of clinical doctors. As is well known, developing countries, such as China, often lag behind developed countries in the cultivation of medical equipment and medical talents. The demand and supply of healthcare are severely imbalanced. In the future, AI models entering clinical practice can serve as an auxiliary tool for preoperative risk stratification in CC patients, improving the diagnostic efficiency of radiologists, avoiding the harm caused by pathological examinations, reducing medical burden, providing greater medical supply, and easing doctor-patient conflicts. However, the differences in imaging equipment and the robustness of AI model construction pose serious challenges to the sustainability of AI entering clinical practice. At present, developing countries need to invest more computer equipment and scientific research to further optimize and update AI technology, enhance AI computing intelligence, and overcome various problems faced by equipment and model development processes.

### 4.3 Limitations

This review encountered several limitations that were noteworthy (1): the relatively small number of studies included for DOI and LVSI evaluations could not entirely exclude the possibility of overzealous exclusion during the literature screening process. Consequently, the generalizability of the meta-analysis results remained a pertinent discussion topic. (2) the meta-analysis results revealed that the overall SENS and SPEC were highly heterogeneous. This heterogeneity primarily stemmed from the lack of standardization in scientific rigor and clinical relevance. (3) The use of the Risk of Bias in RQS and QUADAS-2 tool had evaluation limitations, resulting in controversial interpretations. (4) All studies were retrospective studies, lacking the validation of AI’s effectiveness through high-quality, multicenter, prospective studies.

## 5 Conclusions

This system review and meta-analysis indicates that preoperative R based on MRI images can effectively evaluate the DOI, LVSI, and LNM status in uterine cancer. Moreover, R combined with C factors can improve evaluation performance. However, it is acknowledged that the current research on risk assessment is heterogeneous and thus requires further development and refinement to achieve the goal of facilitating preoperative clinical diagnosis and treatment decisions with precision.

## Data availability statement

The original contributions presented in the study are included in the article/**Supplementary Material**. Further inquiries can be directed to the corresponding author.

## Author contributions

LW: Conceptualization, Investigation, Methodology, Project administration, Software, Visualization, Funding acquisition, Writing – original draft, Writing – review & editing. SoL: Investigation, Methodology, Software, Visualization, Writing – original draft, Writing – review & editing. ShL: Data curation, Formal analysis, Methodology, Validation, Writing – original draft, Writing – review & editing. YL: Investigation, Methodology, Software, Validation, Visualization, Writing – original draft, Writing – review & editing. DW: Data curation, Project administration, Resources, Supervision, Writing – original draft, Writing – review & editing.
